# Psychological correlates of Irritable Bowel Syndrome: Evidence from a comparative study in a Greek sample

**DOI:** 10.1192/j.eurpsy.2026.11040

**Published:** 2026-07-31

**Authors:** A. Karoumpa, A. Papadopoulou, V. Svolos, E. Vousoura, P.-D. Stavrou, V. Efstathiou

**Affiliations:** 1 Department of Psychology, National and Kapodistrian University of Athens; 2 Second Department of Psychiatry, “Attikon” University General Hospital, National and Kapodistrian University of Athens, Athens; 3 Laboratory of Clinical Nutrition and Dietetics, Department of Nutrition and Dietetics, University of Thessaly, Trikala, Greece

## Abstract

**Introduction:**

Irritable Bowel Syndrome (IBS) is a chronic and relapsing disorder of brain–gut interaction, often accompanied by considerable psychological distress. Prior research has linked IBS with elevated anxiety, depression, and pain, as well as reduced self-esteem and well-being. Yet, the complex interconnections among these psychological factors -and how they jointly influence well-being- remain poorly understood.

**Objectives:**

The study aimed to (a) examine differences between individuals with and without IBS in terms of anxiety, depression, pain, self-esteem, and well-being; (b) explore the role of demographic (age, gender) and clinical factors (relapses, therapeutic approach, age of diagnosis, surgical interventions) in psychological outcomes; (c) identify independent correlates of well-being among IBS patients; and (d) investigate the potential mediating role of self-esteem in the relationship between depression and well-being.

**Methods:**

The sample comprised 239 adults (123 with IBS and 116 controls without gastrointestinal disorders). Participants completed the Hospital Anxiety and Depression Scale (anxiety and depression), the Mental Health Continuum-Short Form (mental well-being), the McGill Pain Questionnaire-Short Form (perceived pain), the Rosenberg Self-Esteem Scale (self-esteem), and a demographic and clinical questionnaire. Statistical analyses included parametric and non-parametric tests, correlation analyses, and analysis of covariance to control for confounders. Mediation models were applied to assess the direct and indirect effects of depression on well-being, with self-esteem examined as a potential protective factor.

**Results:**

Participants with IBS reported significantly higher levels of anxiety, depression, and pain, and lower levels of self-esteem and well-being compared to the control group, with elevated rates of probable clinical anxiety and depression. These differences remained significant after adjusting for age. Among IBS patients, flare status and number of relapses were linked to greater psychological burden, while prior gastrointestinal surgery was associated with more pain. In multivariable analyses, well-being was independently related to lower depression, higher self-esteem, female gender, and older age at diagnosis. Mediation analysis further showed that self-esteem partially mediated the association between depression and well-being, underscoring its potential protective role.

**Conclusions:**

IBS was associated with substantial psychological burden, adversely affecting quality of life and mental well-being. Depression and self-esteem emerged as significant correlates of well-being, while self-esteem partly buffering the adverse impact of depression. These findings underscore the need for a holistic, interdisciplinary approach to IBS management, integrating medical and psychological care to promote comprehensive treatment and improve quality of life.

**Disclosure of Interest:**

None Declared

